# Antifungal, anti-inflammatory and cytotoxicity activities of three varieties of *labisia pumila* benth: from microwave obtained extracts

**DOI:** 10.1186/1472-6882-13-20

**Published:** 2013-01-24

**Authors:** Ehsan Karimi, Hawa ZE Jaafar, Syahida Ahmad

**Affiliations:** 1 Department of Crop Science, Faculty of Agriculture, University Putra Malaysia, Serdang, Selangor, 43400 UPM, Malaysia; 2 Department of Biochemistry, Faculty of Biotechnology and Biomolecular Sciences, University Putra Malaysia, Serdang, Selangor, 43400 UPM, Malaysia

## Abstract

**Background:**

*Labisia pumila,* locally known as Kacip Fatimah, is a forest-floor plant that has tremendous potential in the herbal industry. It is one of the five herbal plants identified by the government as one of the national key economic areas to be developed for commercial purposes. There are three varieties of *L. pumila* namely, *L. pumila* var. *pumila*, *L. pumila* var. *alata* and *L. pumila* var. *lanceolata* and each has its own use.

**Methods:**

The leaves and roots of the three varieties of *L. pumila* Benth. were extracted using microwave assisted extraction (MAE). Antifungal activity of all plant extracts were characterized against *Fusarium* sp., *Candida* sp. and *Mucor* using the agar diffusion disc. Anti-inflammatory assays were performed using NO production by macrophage RAW 264.7 cell lines induced by LPS/IFN-g and cytotoxic activity was determined using several cancer cell lines and one normal cell line.

**Results:**

The overall result demonstrated that leaf and root extracts of all three varieties of *L. pumila* exhibited moderate to appreciable antifungal activity against *Fusarium* sp., *Candida* sp. and *Mucor* compared to streptomycin used as positive control. Leaf and root extracts of all varieties significantly decreased NO release. However, the root extracts showed higher activity compared to the leaf extracts. Cytotoxic activity against MCF-7, MDA-MB-231 and Chang cell lines were observed with all extracts.

**Conclusions:**

These findings suggest the potential use of *L. pumila* Benth. as a natural medicine and indicated the possible application of this medicinal plant such anti inflammatory activity and cytotoxic agents.

## Background

Currently, there is growing interest in the application of plants as a medicinal agent since synthetic drugs have shown several side effects on the human body. Experimental investigations demonstrated that many naturally occurring agents in plant extracts have shown antioxidant, antimicrobial and anticancer potential in a variety of bioassay systems and animal models, having relevance to human disease [1]. Medicinal plants are known to have weak or strong therapeutic abilities and contribute in reducing risk of diseases of various etiologies such as inflammatory and cancer. This is attributed to the large amounts of phytoconstituents such as flavonoids, phenolics and saponins [2] found in medicinal plants. These bioactive compounds have received considerable attention due to their therapeutic potential for antimicrobial, anti-inflammatory, anticancer and antioxidant activities [3]. Burda and Oleszek [4] had demonstrated high antiradical activity of some flavonoids like rutin, kaempferol, morin and fustin as strong scavengers with radical scavenging activity of more than 90%. Faried *et al.*[5] indicated that gallic acid isolated from *Phaleria macrocarpa* induced cancer cell death in various cancer cells such as breast cancer (MCF-7), gastric cancer (MKN-28) and colon cancer (HT-29, colon 201 and colon 26). *Labisia pumila* Benth. (Myrsinaceae) popularly known as Kacip Fatimah, is a herbaceous plant with creeping stems used by Malay women to induce and facilitate childbirth as well as a post-partum medicine. Recently, it was found that the bioactive compounds of *L. pumila* consisted of resorcinols, flavonoids and phenolic acids. These compounds have been identified as natural bioactive compounds with high biological activity [6]. Stone [7] had categorized three varieties of this herb in Malaysia namely *L. pumila* (Blume) var. *alata*, *L. pumila* (Blume) var. *pumila* and *L. pumila* var. *lanceolata*. Each of these varieties has different applications. Uses of this herb include treatment for dysentery, dysmenorrheal, flatulence, and gonorrhea [8]. The results of our previous experiments revealed that all three varieties of *L. pumila* Benth. consisted of bioactive compounds including phenolics, flavonoids and saponins which might be responsible for the enhanced antioxidant activity observed in this plant [9]. This present study was carried out to evaluate antifungal, anti-inflammatory and cytotoxic activity of leaf and root extracts derived by microwave extraction from the three varieties of *Labisia pumila* Benth.

## Methods

### Plant materials

Seedlings of the three *Labisia pumila* varieties were collected from places of origin at Hulu Langat (Selangor), Sungkai (Perak) and Kota Tinggi (Johore), respectively, and raised under glasshouse conditions for 18 months before use in the study. Voucher specimens were identified by the Herbarium unit, Institute of Bioscience, University Putra Malaysia (*alata* (Stone 6030 (KLU)), *pumila* ((Stone 7233 (KLU)), and *lanceolata* ((Stone 8385 (KLU)). Healthy and uniform seedlings in terms of leaf numbers were selected from the three varieties. The leaves and roots of selected seedlings were cleaned, separated, freeze dried and stored until further analysis.

### Microwave assisted extraction (MAE)

MAE was performed using a microwave apparatus with a closed vessel system under pressure (ETHOS® T Microwave digestion/extraction system, Milestone Co., Italy) based on the method described by Xiao *et al.*[10] with some modifications. One gram of leaf and root samples of the three varieties of *L. pumila* was weighed in a clean aluminum container, and then transferred into the vessel of the Ethos E Microwave Extraction System and extracted with 30 ml of methanol for 2 min (p = 750 w). The extraction temperature was 60°C. After extraction, the vessels were allowed to cool to room temperature, and the extracts were filtered and stored in a refrigerator.

### Antifungal activity assay

The antifungal assay was carried out by the agar well diffusion method [11] with slight modifications. Briefly, a suspension of the tested fungi was prepared (10^5^ spore/mL) and dispensed (100 μL) uniformly on the surface of the agar plate. Small wells were cut in the agar plate using a cork borer (6 mm). A fixed volume of the different extracts or amphotericin B (PAA Lab., Germany) as positive control (at 450 μg/well), were loaded into the wells. The plates were incubated at 29°C for 72 h. The diameter of the inhibition zone around each well was then recorded in four different directions.

### Anti-inflammatory assay

The murine monocytic macrophage cell line RAW 264.7 was cultured in Dulbecco’s Modified Eagle Media (DMEM) (2mM L-glutamine, 45 g/L glucose, 1 mM sodium pyruvate, 50 U/ml penicillin; 50 μg/ml streptomycin) with 10% foetal bovine serum (FBS). The cells were cultured at 37°C with 5% CO_2_ and were split twice a week. Approximately 1 × 10^6^ cells/ml of RAW 264.7 cells were seeded in 96-well tissue culture plates and incubated for 24 h at 37°C with 5% CO_2_. The cells were then incubated in prepared DMEM medium containing 100 μl of test extract in DMSO and serially diluted, to give a final concentration of 100 μg/ml in 0.1% DMSO. Cells were then stimulated with 200 μ/ml of IFN-γ and 10 μg/ml LPS for 17 h. The presence of nitrite in the cell culture media was determined using Griess reagent and absorbance was read at 550 nm using the microplate reader (Spectra Max Plus 384, Molecular Devices Inc., USA). Nitrite concentration in the supernatants was determined by comparison with a sodium nitrite standard curve. The cell viability was detected by MTT cytotoxicity assay. L-NAME was used as iNOS inhibitor (control) at a concentration 250 μM [12].

### Anti cancer activity assay

Human cancer cell lines (MCF-7; MDA-MB-231) and Human hepatocytes (Chang liver cells) cell lines obtained from the American Type Culture Collection (ATCC) were used in this study. Cells were grown at 37°C in humidified 5% CO_2_ and 95% air atmosphere in Dulbecco’s Modified Eagle Media (DMEM) (2mM L-glutamine, 45 g/l glucose, 1 mM sodium pyruvate, 2g/l sodium bicarbonate and 10% fetal bovine serum). Monolayers of cells (5 × 10^3^/100 μl) were grown in 96-well microlitre plates and exposed to two fold serial dilutions of the extracts from 200 μg to 3.1 μg/100 μl. After 3 days incubation at 37°C, the cytotoxicity of extracts was determined using the MTT assay according to Ahmad *et al.*[12]. Tamoxifen, an anticancer drug, was used as the positive control in the present study.

### Statistical analysis

Data were analyzed using the analysis of variance procedure in the Statistical Analysis System (SAS) Version 9 (SAS Institute, Cary, NC). Significant differences between means from triplicate analyses (p < 0.05) were determined by Duncan’s Multiple Range Test. GraphPad Prism 5 software (GraphPad Software Inc., San Diego, CA) was also used in data analyses.

## Results and discussion

### Antifungal activity of the three varieties of *Labisia pumila* Benth

The results of antifungal activity of leaf and root extracts from the three varieties of *Labisia pumila* Benth against three fungi species are summarized in the Table 1. In general, most of the extracts assayed exhibited weak to moderate inhibitory activity against all fungi tested. The leaves compare to roots exhibited higher activity towards the tested fungi at a concentration of 450 μg/well in all three varieties. The highest inhibitory activity was observed in the leaf extract of variety *pumila* against *Candida* sp. (inhibition zone = 0.82 cm) and leaf extract of variety *alata* against *Mucor* sp. (inhibition zone = 0.70 cm). The lowest inhibitory activity was obtained with the root extract of variety *lanceolata* against *Mucor* sp. (inhibition zone = 0.33 cm). Antifungal activity of all the extracts is attributed to presence of bioactive compounds such as flavonoids, phenolics, saponins and various volatile compounds [13]. Various extracts of medicinal plants have also been shown to have inhibitory effects against phytopathogenic fungi *in vitro*[14]. Vaquero *et al.*[15] revealed that different concentrations of gallic acid, caffeic acid, rutin and quercitin of wine exhibited various sensitivities against pathogenic fungi.

**Table 1:** **Inhibition zones of leaf and root extracts of three varieties of *Labisia pumila *against pathogenic fungi at a concentration of 450 μg/well**

*Sample		**Inhibition Zone (diameter in cm)**	
	***Fusarium *sp.**	***Candida *sp.**	***Mucor *sp.**
*Pumila* Leaf	0.65^c^	0.82^b^	0.70^c^
*Alata* Leaf	0.71^b^	0.75^c^	0.81^b^
*Lanceolata* Leaf	0.45^g^	0.67^d^	0.57^d^
*Pumila* Root	0.60 ^d^	0.55^f^	0.47^e^
*Alata* Root	0.57 ^e^	0.62^e^	0.45^f^
*Lanceolata* Root	0.49 f	0.51^g^	0.33^g^
Streptomycin	1.28^a^	1.24^a^	1.18^a^

Means with different superscripts within column are significantly different (P < 0.05).

* Sample - methanolic extracts and standard antibiotic agent.

### Anti-inflammatory activity of the three varieties of *Labisia pumila* Benth

**Figure 1:**
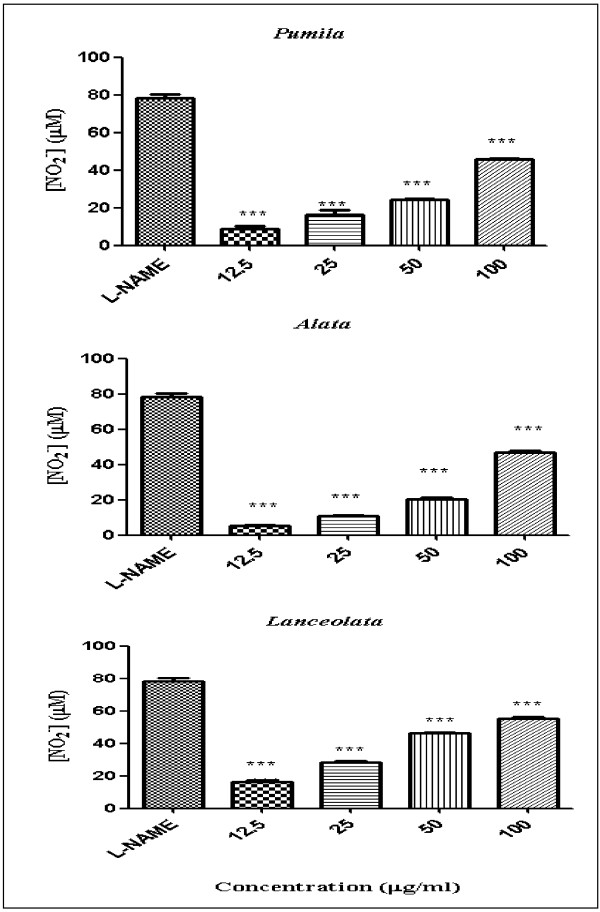
Percentage NO inhibition with *L. pumila *var. *pumila, alata and lanceolata *leaf extracts [All values represent mean ± standard deviation of three different experiments; ***p < 0.001, **p < 0.01 and *p < 0.05 indicate significant difference compared to the control (L-NAME)].

**Figure 2:**
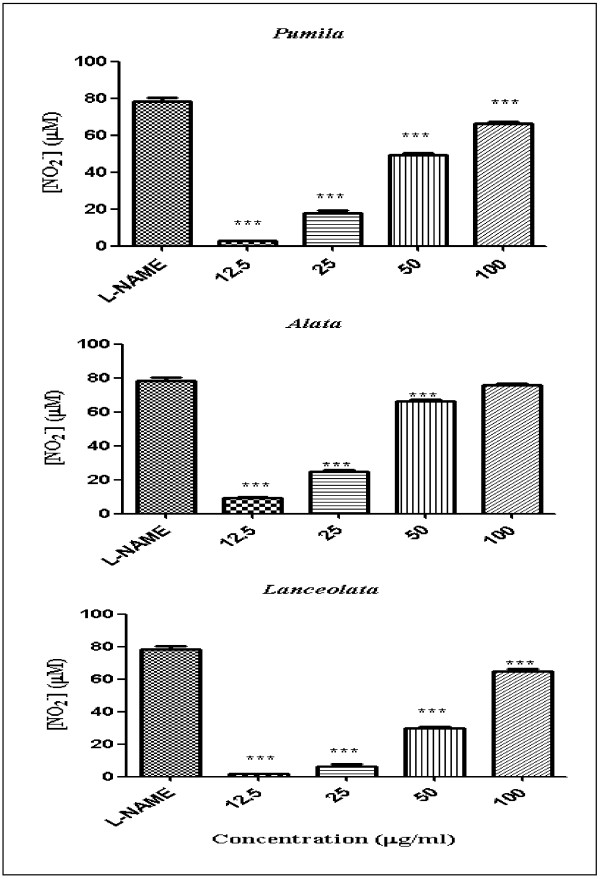
**Percentage NO inhibition with *L. pumila *var. *pumila, alata and lanceolata *root extracts [All values represent mean ± standard deviation.** ***p < 0.001, **p < 0.01 and *p < 0.05 indicate significant difference compared to the control (L-NAME)].

**Figure 3:**
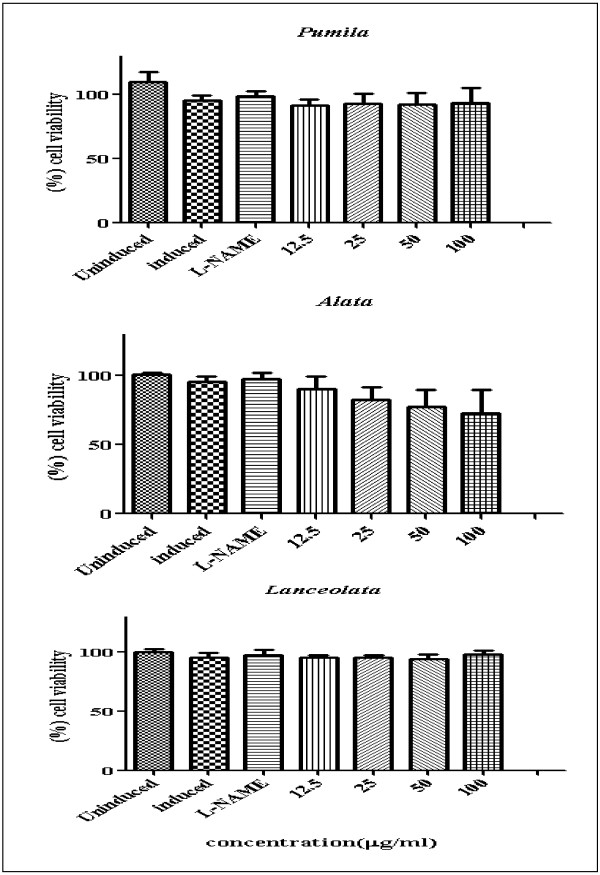
**Effect of leaf extracts of *Labisia pumila *var. *pumila*, alata and *lanceolata *on viability of RAW 264.7 cells [Each bar represents the mean ± standard deviation of three independent experiments].**

**Figure 4:**
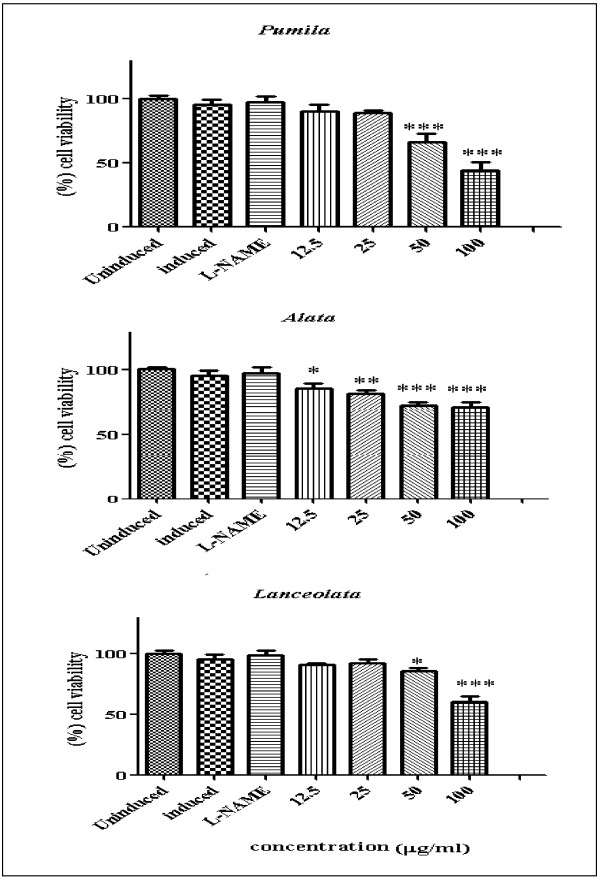
**Effect of root extracts of *Labisia pumila *var. *pumila*, alata and *lanceolata *on viability of RAW 264.7 cells [Each bar represents the mean ± standard deviation of three independent experiments].**

Nitric oxide (NO) is an important radical molecule that is produced by a family of enzymes known as nitric oxide synthase (NOS) and participates in the physiology and pathophysiology of many systems. Producing large amount of NO is associated with numerous pathological conditions. NO released from cells can be detected and quantified photometrically as its stable product, nitrite in the cell culture supernatant [16]. Anti-inflammatory activity of leaves and roots of the three varieties of *L. pumila* was evaluated based on the colorimetric nitric oxide assay. In this study, all extracts were examined for ability to inhibit NO release in RAW 264.7 cells stimulated with bacterial lipopolysaccharides (LPS) and interferon-gamma (IFN-γ). All extracts were assayed at the two-fold dilution range (12.5-100 μg/ml) dissolved in 0.2% DMSO and compared with N-Nitro-L-Arginine Methyl Ester (L-NAME). The viability of RAW 264.7 cells after treatment was assessed by the MTT (3–4,5- dimethylthiazol-2-yl)-2,5-diphenyl tetrazolium bromide) method. The activity profiles of the extracts in terms of percentage of NO inhibition were categorized according to Kim *et al.*[17]. Inhibition of NO production in LPS/IFN-γ stimulated RAW 264.7 cell lines were classified into four ranks which are: strongly active (70% and above); moderately active (50-69%); weakly active (30-49%); and very weakly active (29% or less). Figures 1 and 2 show the NO inhibition of L-NAME, leaf and root extracts of the three varieties of *L. pumila* (var. *pumila, alata* and *lanceolata*). L-NAME inhibited NO production by 78.43%. The NO inhibition values of leaf extract of *L. pumila* var. *pumila, alata* and *lanceolata* were 45.66, 46.64 and 55.17%, respectively, at a concentration of 100 μg/ml, while the root extract of var. *pumila, alata* and *lanceolata* resulted in 66.45, 75.68 and 64.81%, inhibition, respectively (Table 2). The results show that all the extracts have weak to moderate activity compare to control (Table 2). MTT assay was also performed to test the cell viability of RAW 264.7 cell lines. This step is crucial to prove that the inhibition of NO production was not false due to cell death. The cell viability with L-NAME, leaf and root extracts are shown in Figures 3 and 4. All the extracts showed moderate activity and the percentage of cell viability was affected (Table 1). At the highest concentration used (100 μg/ml) percentage cell viability for each leaf and root extract of *L. pumila* was more than 65%. The results suggest that leaf and root extracts of *L. pumila* appeared to be potent iNOS inhibitors contributing to anti-inflammatory effects and at the same time were non-toxic to Raw 264.7 cells. The potential of *L. pumila* Benth as an anti-inflammatory agent is attributed to the presence of phytochemicals such as flavonoids and phenolics as well as saponins. Karimi *et al.*[9] showed that kaempferol, naringin and myricetin were the main flavonoid compounds present in all three varieties with values of 222.8, 79.4, 31.1 μg/g dry sample in the leaves of var *pumila*, 187.2, 140, 88.4 μg/g dry sample of var *alata,* and 164.6, 86.7, 27.9 μg/g dry sample of var *lanceolata*, respectively. The analyses also confirmed the presence of gallic acid and caffeic acid as the main phenolic compounds in all three varieties. It was also noted that all three varieties of *L. pumila* Benth had high saponin contents in their leaves and roots. The mechanism of phenolic compounds in antioxidant activity and their ability to act as free radical and NO scavengers, leading to the formation of phenoxyl radicals have been described by Sumanont *et al.*[18]. Oskoueian *et al.*[19] suggested that the inhibition of iNOS in the RAW 264.7 cell is due to the suppressive action of flavonoids. Furthermore, saponins isolated from roots of *Physospermum verticillatum* exerted significant inhibition of NO production in LPS induced RAW 264.7 macrophages [20]. Meanwhile, *in vitro* studies have demonstrated that phytochemicals are able to suppress iNOS and inhibit nitric oxide and this ability depends on the structure of compounds. Kim *et al.*[21] reported that iNOS, an enzyme-catalyzing NO production, was found to be over-expressed in chronic inflammatory diseases and various types of cancer. NO is an important regulatory molecule in inflammation response and cancer development. Under the circumstances of chronic inflammation, the continuous generation of NO may lead to DNA damage, disruption of DNA repair, and cancer prone post-translational modification.

**Table 2:** **Effect of leaf and root extracts of three varieties of *L. pumila *Benth. on NO production and cell viability of RAW 264.7 cells**

**Sample Extract**	**Level of Activity**	**NO inhibition (%)**^**a**^	**Cell viability (%)**^**a**^
*Pumila* Leaf	weak	45.66 ± 1.29	93.64 ± 11.59
*Alata* Leaf	weak	46.64 ± 2.10	72.75 ± 17.06
*Lanceolata* Leaf	moderate	55.17 ± 1.96	98.12 ± 3.71
*Pumila* Root	moderate	66.45 ± 1.84	44.57 ± 6.06
*Alata* Root	strong	75.68 ± 1.70	71.32 ± 4.26
*Lanceolata* Root	moderate	64.81 ± 2.22	60.26 ± 4.56
L-NAME (Control)	strong	78.43 ± 0.97	97.67 ± 4.40

The data represent means ± standard deviation of triplicate cultures of three independent experiments.

^a^ Values expressed at concentration 100 μg/ml.

### Anticancer activity of the three varieties of *Labisia pumila* Benth

**Figure 5:**
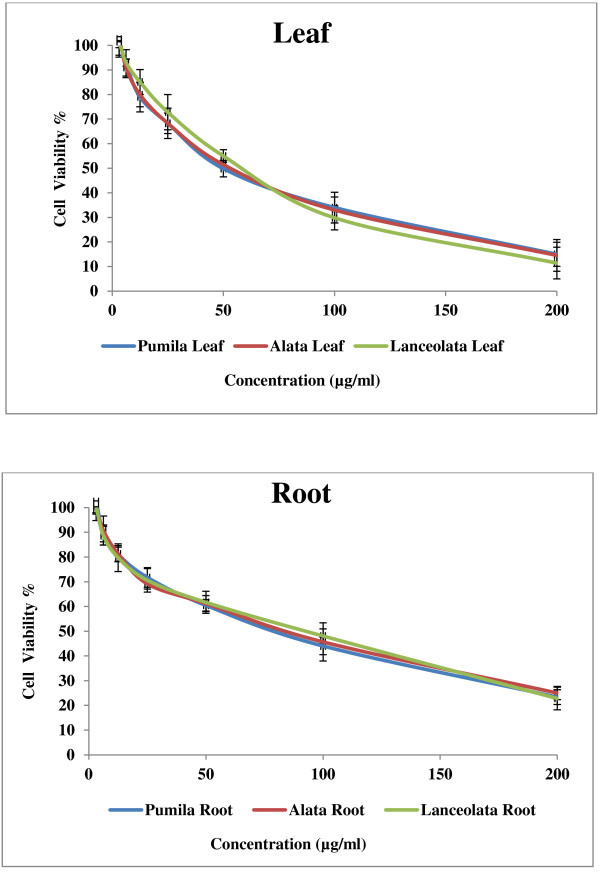
**Effect of leaf and root extracts of *Labisia pumila *var. *pumila*, *alata *and *lanceolata *on MCF-7 cell viability [All values represent the mean ± standard deviation from three independent experiments].**

**Figure 6:**
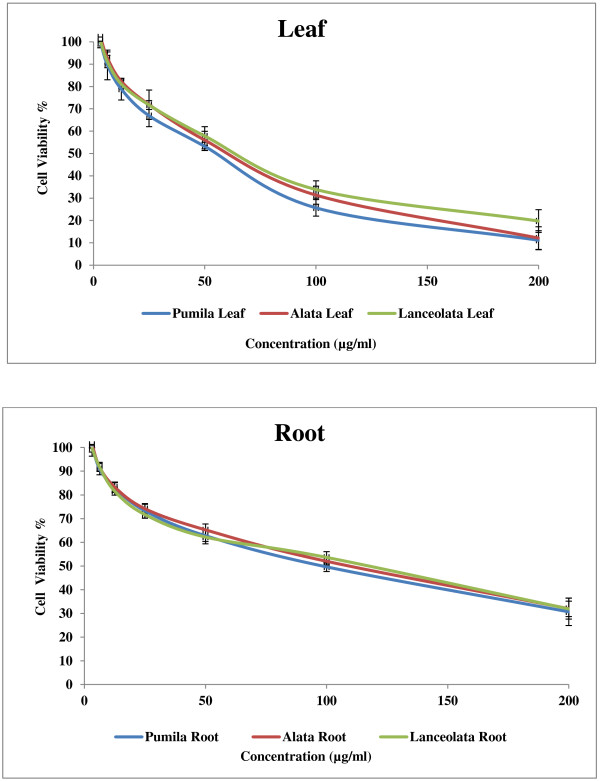
**Effect of leaf and root extracts of *Labisia pumila *var. *pumila*, *alata *and *lanceolata *on MDA-MB 231 cell viability [All values represent the mean ± standard deviation from three independent experiments].**

**Figure 7:**
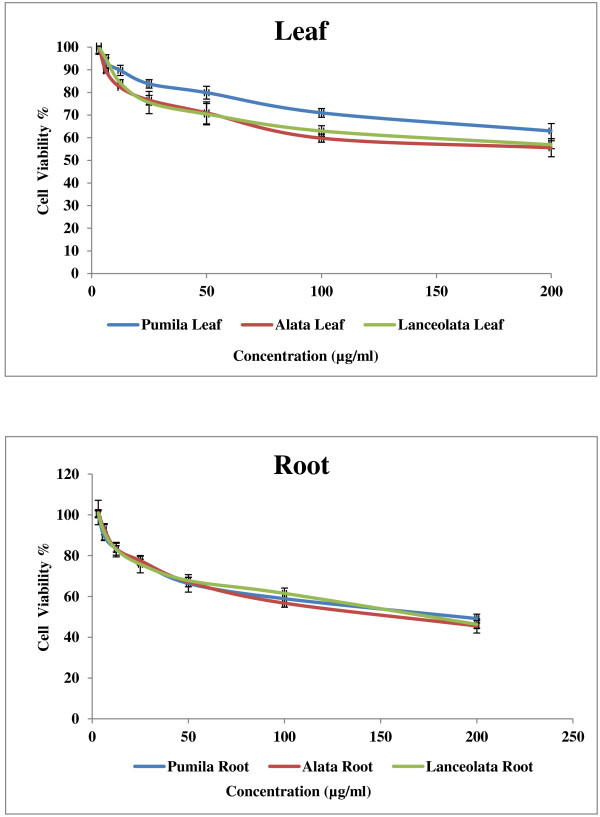
**Effect of leaf and root extracts of *Labisia pumila *var. *pumila*, *alata *and *lanceolata *on CHANG cell viability [All values represent the mean ± standard deviation from three independent experiments].**

**Figure 8:**
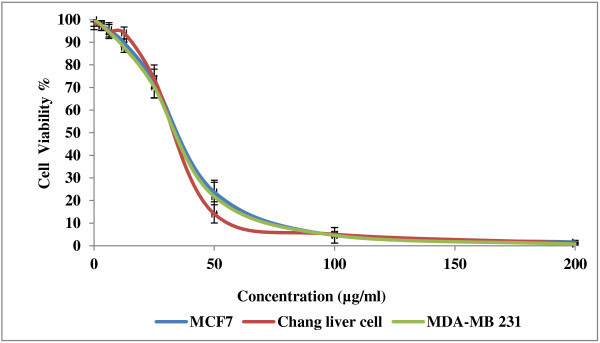
Effect of Tamoxifen on MCF7, Chang liver and MDA-MB 231 cell viability [All values represent the mean ± standard deviation from three independent experiments].

Cancer is a group of diseases characterized by cells that grow out of control. In most cases, they form masses of cells, or tumors, that infiltrate, crowd out, and destroy normal tissue. Consumption of antioxidant rich fruits and vegetables in our daily diets significantly reduces the risk of many cancer diseases suggesting that antioxidants could be effective agents for the inhibition of cancer spread [22]. The results of anticancer activity of leaf and root extracts of the three varieties of *Labisia pumila* Benth are presented in Figures 5, 6 and 7. Increase in extract concentrations of upto 200 μg/ml reduced the cell viabilities significantly (p < 0.001) in a dose-dependent manner in all three cell lines tested. The IC_50_ values of extracts used in this study are presented in Table 3. According to the US NCI plant screening program, a crude extract is generally considered to have *in vitro* cytotoxic activity if the IC_50_ value (concentration that causes reduction in cell viability to 50%) was less than 30 μg/ml [23]. Leaf extracts appeared to be more active compared root extracts for all varieties on the three cell lines tested. *Labisia pumila* var. *pumila* exhibited more cytotoxic activity compared to var. *alata* followed by var. *lanceolata*. The IC_50_ concentrations for leaf extracts of *L. pumila* var. *pumila*, *alata* and *lanceolata,* with the MCF7 cell line were found to be 48.2, 54 and 59.9 μg/ml and for MDA-MB-231 the values were 55.80, 62.26 and 66.39 μg/ml, respectively. In terms of toxicity to the Chang liver cells, leaf and root extracts were considered as not toxic as the IC_50_ values were greater than 200 μg/ml. Tamoxifen was used as the positive control in this study (Figure 8). The IC_50_ concentrations of Tamoxifen for Chang liver cell, MCF-7 and MDA-MB-231 were 34.97, 36.54 and 35.46 μg/ml, respectively (Table 3). The activity of Tamoxifen was found to be higher than the leaf and root extracts. Tamoxifen has been used specifically to treat breast cancer due to its antagonistic effect on estrogen receptors in breast tissue [24]. All leaf and root extracts showed anticancer activity at various concentrations. This may be attributed to the different bioactive compounds such as phenolics and flavonoids that could lead to the cytotoxic activity of this medicinal plant. Chang liver cell was also susceptible to the extracts indicating the lack of selectivity in the effect. The GC-MS analysis of methanolic extracts reported by Karimi *et al.*[13] demonstrated more than 25 compounds in the leaves of the three varieties of *Labisia pumila* Benth. Heptadecanoic acid (20.39%), octadecanoic acid (16.24%) and 1,3-dioxolane,2,4,5-trimethyl (18.69%) were the main compounds in the leaf extracts of *Labisia pumila* var. *alata*, *pumila* and *lanceolata*. These compounds have been reported to have antibacterial and antifungal [25], antioxidant [26] and anticancer activities [27]. It is also known that high concentration of certain fatty acids can cause cell death via apoptosis or necrosis [28]. Oskoueian *et al.*[19] indicated the ability of flavonoids and phenolic compounds to act as anticancer agents. Zhang *et al.*[29] showed that kaempferol, quercetin, anthocyanins, coumaric acid and ellagic acid isolated from strawberry inhibited the growth of human cancer cell lines for breast (MCF-7), oral (KB, CAL-27), colon (HT-29, HCT-116), and prostate (LNCaP, DU-145). Similar results have also been reported in previous studies where polyphenols such as resveratrol, quercetin, catechin, and epicatechin isolated from wine extract [30] and green tea polyphenols like epigallocatechin and epicatechin [31] play an important role as anticancer agents.

**Table 3:** **IC**_**50**_**values of leaf and root extracts of *L. pumila *Benth on Chang liver cells and MCF-7 and MDA-MB-231 cell lines**

**Sample**	**IC**_**50**_**Value (μg/ml)**		
	**Chang liver cell**	**MCF-7**	**MDA-MB-231**
*Pumila* Leaf	> 200	48.19 ± 2.59	55.80 ± 1.83
*Alata* Leaf	> 200	53.96 ± 3.41	62.26 ± 2.92
*Lanceolata* Leaf	> 200	59.88 ± 2.63	66.39 ± 2.74
*Pumila* Root	192.16 ± 1.48	81.92 ± 2.28	98.93 ± 1.93
*Alata* Root	160.90 ± 2.15	86.14 ± 2.11	110.30 ± 1.52
*Lanceolata* Root	176.68 ± 0.97	92.94 ± 2.54	116.87 ± 2.41
Tamoxifen (control)	34.97 ± 1.55	36.54 ± 0.79	35.46 ± 0.46

All analyses were mean of triplicate measurements ± standard deviation.

## Conclusion

Medicinal plants are now increasingly getting more attention than ever before. The medicinal value of these plants lies in the bioactive phytochemical constituents such as phenolics, flavonoids and saponins that result in various biological activities [32]. The results of this study showed the potential of *Labisia pumila* plants and in particular the leaves for use in the development of anti fungal, anti-inflammatory and anti cancer drugs using microwave extraction. These activities might be due to the presence of various phenolic and flavonoid compounds in the leaves compare to root in all three varieties of *L. pumila* Benth [9,13]. It should be noted that the results obtained were based on *in vitro* screening tests. Therefore isolation of bioactive compounds responsible for the observed activities is necessary and *in vivo* studies are needed for further confirmation.

## Competing interests

The authors declare that they have no conflicts of interest concerning this article.

## Authors’ contributions

EK conducted antifungal properties, cytotoxicity assay, analyze and interpretation of data, and drafted the manuscript. HZEJ was responsible for conception and design, drafted the manuscript and revised it critically for important intellectual content. SA conducted the anti-inflammatory assay, and assisted in the analysis and interpretation of data. All authors read and approved the final manuscript.

## Pre-publication history

The pre-publication history for this paper can be accessed here:

http://www.biomedcentral.com/1472-6882/13/20/prepub
